# Диагностика психогенной полидипсии на примере клинического случая

**DOI:** 10.14341/probl13359

**Published:** 2023-09-21

**Authors:** Э. Б. Казангапов, Л. Н. Бельчикова

**Affiliations:** Тюменский государственный медицинский университет; Тюменский государственный медицинский университет

**Keywords:** несахарный диабет, первичная полидипсия, десмопрессин, тест с водной депривацией

## Abstract

Полидипсия — это патологически усиленная жажда, удовлетворяемая приемом воды в больших количествах, которая может проявляться при разных соматических или психических заболеваниях и, на первый взгляд, имеет сходную клиническую картину с истинным дефицитом вазопрессина.

Центральный несахарный диабет (ЦНД) — заболевание гипоталамо-гипофизарной области, характеризующееся неспособностью почек реабсорбировать воду и концентрировать мочу, в основе которого находится дефект синтеза или секреции вазопрессина, и проявляющееся выраженной жаждой и экскрецией большого количества гипотоничной мочи. Распространенность заболевания в популяции составляет 1:25 000, что характеризует его как достаточно редкую патологию гипоталамо-гипофизарной области. Пик заболеваемости приходится на 30–40 лет. Согласно разным литературным источникам, заболевание не характеризуется гендерными различиями в распространенности, однако на примере московской популяции в структуре заболеваемости ЦНД преобладали женщины в соотношении 2,2:1. Патологическое значение первичной полидипсии заключается в проявлениях водной интоксикации, тем самым это состояние требует знания четких диагностических критериев специалистами здравоохранения и междисциплинарного подхода в его терапии.

На примере этого клинического случая мы постараемся выделить дифференциально-диагностические критерии психогенной полидипсии в сравнении с истинным дефицитом аргинин-вазопрессина (АВП) или центральным несахарным диабетом (ЦНД), которые можно применять в клинической практике.

## Abstract (en)

Polydipsia is a pathologically increased thirst, satisfied by the intake of water in large quantities, which can manifest itself in various somatic or mental diseases and at first glance is similar to a true vasopressin deficiency. Central diabetes insipidus (CDI) is a disease of the hypothalamic-pituitary region characterized by the inability of the kidneys to reabsorb water and concentrate urine, which is based on a defect in the synthesis or secretion of vasopressin and is manifested by severe thirst and excretion of large amounts of hypotonic urine. The prevalence of the disease in the population is 1:25,000, which characterizes it as a fairly rare pathology of the hypothalamic-pituitary region. The peak incidence is between 30 and 40 years of age. According to various literary sources, the disease is not characterized by gender differences in prevalence, however, on the example of the Moscow population, women prevailed in the incidence structure of CND in a ratio of 2.2:1. Insidiousness, with apparent absences in the difficulty of diagnosing primary polydipsia, lies in the manifestations of water intoxication, thus this condition requires knowledge of clear diagnostic criteria by healthcare professionals and an interdisciplinary approach in the treatment of this condition. On the example of this clinical case, we will try to highlight the differential diagnostic criteria for psychogenic polydipsia in comparison with the true deficiency of arginine vasopressin (AVP) or central diabetes insipidus (CDI), which can be applied in real clinical practice.

## ВВЕДЕНИЕ

Полидипсия — это патологически усиленная жажда, удовлетворяемая приемом воды в больших количествах, которая может проявляться при разных соматических или психических заболеваниях [2] и, на первый взгляд, имеет сходную клиническую картину с истинным дефицитом вазопрессина.

Центральный несахарный диабет (ЦНД) — заболевание гипоталамо-гипофизарной области, характеризующееся неспособностью почек реабсорбировать воду и концентрировать мочу, в основе которого находится дефект синтеза или секреции вазопрессина, и проявляющееся выраженной жаждой и экскрецией большого количества гипотоничной мочи [1][3][5]. Распространенность заболевания в популяции составляет 1:25 000, что характеризует его как достаточно редкую патологию гипоталамо-гипофизарной области. Пик заболеваемости приходится на 30–40 лет. Согласно разным литературным источникам, заболевание не характеризуется гендерными различиями в распространенности, однако на примере московской популяции в структуре заболеваемости ЦНД преобладали женщины в соотношении 2,2:1 [1][4][5]. Патологическое значение первичной полидипсии заключается в проявлениях водной интоксикации, тем самым это состояние требует знания четких диагностических критериев специалистами здравоохранения и междисциплинарного подхода в его терапии.

На примере этого клинического случая мы постараемся выделить дифференциально-диагностические критерии психогенной полидипсии в сравнении с истинным дефицитом аргинин-вазопрессина (АВП) или центральным несахарным диабетом (ЦНД), которые можно применять в клинической практике.

## ОПИСАНИЕ КЛИНИЧЕСКОГО СЛУЧАЯ

История заболевания. Женщина, 67 лет, поступила в эндокринологическое отделение ГБУЗ ТО «Областная клиническая больница №1» г. Тюмени с жалобами на жажду в дневное время, обильное, учащенное мочеиспускание до 4,5 литра в сутки, снижение аппетита, дисфагию, метеоризм, запор, низкие цифры артериального давления, общую слабость, сухость губ и слизистой рта, кожных покровов, снижение слюноотделения, потерю массы тела на 7 кг в течение текущего года. В ходе сбора анамнеза установлено, что впервые данные жалобы появились 2 года назад, когда пациентка отметила обильное, учащенное мочеиспускание. Амбулаторно консультирована урологом с целью исключения патологии мочевыделительной системы, с учетом полиурии до 5 литров в сутки был заподозрен нефрогенный несахарный диабет. По результатам первичного исследования уролога: в микробиологическом исследовании мочи обнаружена E. coli, в исследовании мочи по Зимницкому колебания удельного веса составляли 1005–1012, показатели водно-электролитного обмена (натрий, калий, хлор) находились в пределах референсного диапазона, показатели белкового обмена — без отклонений, нарушения углеводного обмена не верифицированы. По результату сцинтиграфии почек клинически значимой патологии не выявлено. Далее пациентка консультирована нефрологом ГБУЗ ТО «ОКБ №1» г. Тюмени, который установил диагноз «Хронический тубулоинтерстициальный нефрит, ХБП С2 А1», рекомендован прием дипиридамола (в настоящий момент препарат пациентка не принимает в связи с усилением диспепсических явлений), препараты железа. Неоднократно консультирована эндокринологом амбулаторно, который исключал ЦНД, однако функциональные диагностические пробы не проводились в связи с отсутствием технических возможностей для их проведения на амбулаторном этапе, патологий со стороны надпочечников и щитовидной железы не выявлено. По данным МРТ гипофиза от июля 2021 г., аденомы гипофиза не выявлено, патогномоничного для ЦНД гиперинтенсивного сигнала от задней доли гипофиза на Т1 взвешенных не выявлено (рис. 1). В мае 2022 г. пациентка находилась на стационарном лечении в отделении урологии ГБУЗ ТО «ОКБ №2» г. Тюмени с диагнозом: «Острый восходящий пиелонефрит справа», проведен курс антибактериальной терапии. Анализ мочи по Зимницкому: удельный вес — 1005 г/л, суточный диурез — 3470 мл. Повторно проведена консультация эндокринолога, который также исключил дефицит антидиуретического гормона. Из анамнеза жизни известно, что в настоящее время наблюдается у гастроэнтеролога по поводу хронического постописторхозного холецистита, билиарного сладжа, полипа желчного пузыря, хронического панкреатита, долихосигмы, колоноптоза, тубулярной непролиферирующей аденомы толстой кишки. По рекомендации гастроэнтеролога регулярно получает панкреатин 25 000 ЕД х 3 раза в сутки. Данные за перенесенные нейрохирургические вмешательства, черепно-мозговые травмы пациентка отрицает.

**Figure:**
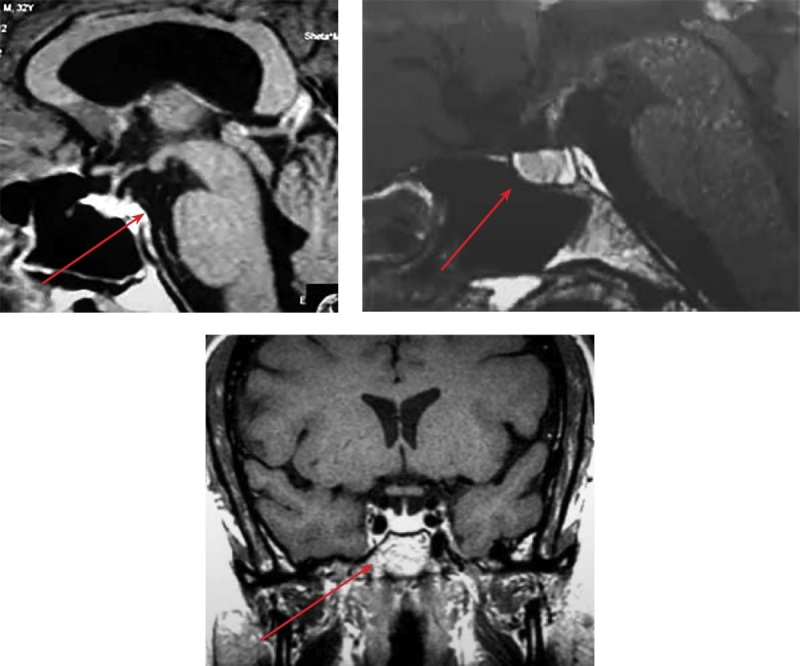
Рисунок 1. МР-данные хиазмально-селлярной области пациента. Турецкое седло в латеральной проекции (слева) и cредняя сагиттальная часть гипофиза и турецкого седла (справа), коронарный срез головного мозга (снизу). Красными стрелками обозначена локализация селлярной области. Figure 1. MR data of the patient's chiasmal-sellar region. The sella turcica in a lateral projection (left) and the middle sagittal part of the pituitary gland and sella turcica (right), coronal section of the brain (bottom). Red arrows indicate the localization of the sellar region.

Объективный статус. Сознание ясное. Положение активное. Масса тела — 68,0 кг. Рост — 172 см. Индекс массы тела — 22,9 кг/м². Температура тела — 36,6 °С. Кожные покровы физиологической окраски, чистые, без особенностей. Склеры обычной окраски. Видимые слизистые оболочки розовые. Зев чистый. Щитовидная железа мягко-эластической консистенции, в размерах не увеличена, узловые образования не пальпируются. Перкуссия и аускультация сердца и легких без особенностей. Артериальное давление — 135/70 мм рт. ст., пульс — 73 уд/мин. Живот при пальпации мягкий, безболезненный.

St. localis: клинических признаков дегидратации не выявлено.

В эндокринологическом отделении запланировано обследование, включавшее клинический минимум (общеклинический анализ крови, мочи, биохимический анализ крови на 13 показателей), исследование осмолярности плазмы крови и мочи, исследование мочи по Зимницкому, УЗИ органов брюшной полости, повторное гормональное исследование (свободный кортизол в слюне, базальный уровень кортизола в плазме крови в 08:00, ТТГ), функциональная проба с депривацией жидкости. Осмоляльность плазмы крови определялась лабораторно, а осмолярность плазмы крови вычислена по формуле:

2*{Na (ммоль/л) + К (ммоль/л)} + глюкоза (ммоль/л) + мочевина (ммоль/л) + 0,03 х общий белок (г/л).

В ходе первичного исследования в отделении значимых отклонений не было выявлено, осмоляльность плазмы составляла 275,3 мОсм/кг, осмолярность плазмы крови по формуле: 285 мОсм/л (референс: 290–320 мОсм/л). Представляем вашему вниманию результат анализа мочи по Зимницкому в таблице 1, а также динамику показателей водно-электролитного обмена в таблице 2.

**Table:** Таблица 1. Результаты анализа мочи по Зимницкому Table 1. Results of urine analysis according to Zimnitsky

Наименование	Результат	Единицы измерения
Объем 1 порции мочи (6–9)	420	мл
Объем 2 порции мочи (9–12)	300	мл
Объем 3 порции мочи (12–15)	650	мл
Объем 4 порции мочи (15–18)	520	мл
Объем 5 порции мочи (18–21)	420	мл
Объем 6 порции мочи (21–24)	отсутствует	мл
Объем 7 порции мочи (0–3)	710	мл
Объем 8 порции мочи (3–6)	500	мл
Относительная плотность 1 порция (6–9)	1 012	г/л
Относительная плотность 2 порция (9–12)	1 009	г/л
Относительная плотность 3 порция (12–15)	1 007	г/л
Относительная плотность 4 порция (15–18)	1 006	г/л
Относительная плотность 5 порция (18–21)	1 005	г/л
Относительная плотность 6 порция (21–24)	отсутствует	г/л
Относительная плотность 7 порция (0–3)	1 005	г/л
Относительная плотность 8 порция (3–6)	1 005	г/л
Дневной диурез	1 890	мл
Ночной диурез	1 630	мл
Суточный диурез	3 520	мл

**Table:** Таблица 2. Динамика показателей водно-электролитного обмена Table 2. Dynamics of water-electrolyte metabolism indicators

Показатели электролитного обмена	Время исследования/результат, ммоль/л	Референсные значения, ммоль/л
Февраль 2023 г.	Май 2023 г.	Июнь 2023 г.
Натрий	138	134	133	130–150
Калий	3,9	5,1	4,8	3,5–5,5
Хлор	103	102	101	95–110

По данным анализа мочи по Зимницкому можно предположить о наличии полиурии (с учетом веса пациентки 68 кг, суточный диурез превышает >40 мл/кг), осмолярность мочи вычислена по формуле [ удельный вес мочи — 1000*33,3] и составляла 166,5 мОсм/кг, однако относительная плотность мочи находилась в пределах 1005–1012 г/л, что несмотря на полученные первичные дискордантные лабораторные данные, являлось показанием для проведения пробы с депривацией жидкости (сухоядением) в соответствии с протоколом G.L. Robertson (2001 г.), которая на сегодня остается «золотым стандартом» для исключения первичной полидипсии. Результаты пробы приведены в таблице 3.

**Table:** Таблица 3. Проба с депривацией жидкости (сухоедением) Table 3. Test with fluid deprivation (dry eating)

Время пробы	Осмолярность плазмы крови (мОсм/кг)	Осмоляльность мочи (мОсм/кг)	Натрий (ммоль/л) (130–150)	Самочувствие	Вес, кг	АД, мм рт.ст. (пульс, уд/мин)
08:00	275,4	375	132	Без отклонений	68	130/88 (69)
09:00	275,2	396	133	Без отклонений	68	125/85 (71)
10:00	275,5	480	135	Незначительная жажда, сухость во рту	67,9	130/80 (70)
11:00	276,3	550	134,7	Незначительная жажда, сухость во рту	68	128/80 (76)
12:00	276,5	598	136,7	Умеренная жажда, сухость во рту	68	131/82 (73)
13:00	276,2	619	137	Умеренная жажда, сухость во рту	68	127/81 (74)
14:00	277,0	623	136,8	Умеренная жажда, сухость во рту	68	125/89 (79)
15:00	276,8	653	137,5	Умеренная жажда, сухость во рту	68	130/85 (77)

Дегидратационный тест был завершен на седьмом часе от начала пробы в связи с повышением осмоляльности мочи до 654 мОсм/кг, ухудшением самочувствия пациента в виде нарастания жажды. За время проведения пробы не выявлено отклонений показателей осмолярности плазмы крови, натриемии и показателей гемодинамики отмечается отсутствие отрицательной динамики массы тела.

Таким образом, по результатам жалоб (появления жалоб на жажду исключительно в дневное время), данных анамнеза, отсутствия отклонений биохимических и гормональных показателей крови и мочи, результатам МРТ-исследования гипофиза и, наконец, повышения осмоляльности мочи до 653 мОсм/кг на фоне пробы с сухоедением позволило исключить диагноз несахарного диабета и верифицировать первичную полидипсию с большой вероятностью на фоне психогенного компонента. По итогам обследования даны рекомендации по лечению сопутствующих заболеваний у гастроэнтеролога и нефролога, а также с учетом установленного диагноза рекомендована консультация психотерапевта для верификации расстройства питьевого поведения. В настоящее время в заместительной терапии препаратами десмопрессина и проведении МРТ-исследований головного мозга пациентка не нуждается с учетом доказанной нозологии.

## ЗАКЛЮЧЕНИЕ

Представленный случай наглядно подчеркивает важность тщательного сбора анамнеза на первичном приеме пациента с инсипидарным синдромом и предполагаемым дефицитом антидиуретического гормона для определения показаний к проведению дегидратационного теста в условиях стационара. Из недостатков описанного клинического случая можно отметить нецелесообразность проведения магнитно-резонансной томографии гипофиза без получения достаточных анамнестических и лабораторных данных, указывающих на наличие у пациента несахарного диабета. На основании описанного наблюдения можно выделить диагностические критерии первичной полидипсии: отсутствие полидипсии в ночное время, никтурии, анамнестических данных о перенесенных нейрохирургических вмешательствах и тяжелых черепно-мозговых травм, отсутствие снижения относительной плотности мочи <1005 г/л и, наконец, повышения осмоляльности мочи на этапе проведения пробы с сухоядением до 650 мОсм/кг и выше после исключения более распространенных причин полиурического синдрома (сахарный диабет, патология фосфорно-кальциевого обмена, нефрологическая и гастроэнтерологическая патологии).

## ДОПОЛНИТЕЛЬНАЯ ИНФОРМАЦИЯ

Источники финансирования. Работа выполнена по инициативе авторов без привлечения финансирования.

Конфликт интересов. Авторы декларируют отсутствие явных и потенциальных конфликтов интересов, связанных с содержанием настоящей статьи.

Участие авторов. Все авторы одобрили финальную версию статьи перед публикацией, выразили согласие нести ответственность за все аспекты работы, подразумевающую надлежащее изучение и решение вопросов, связанных с точностью или добросовестностью любой части работы

Согласие пациента. Пациент добровольно подписал информированное согласие на публикацию персональной медицинской информации в обезличенной форме.
